# Influence of Sintering Temperature on the Marginal Fit and Compressive Strength of Monolithic Zirconia Crowns

**DOI:** 10.30476/DENTJODS.2021.90024.1459

**Published:** 2022-09

**Authors:** Mina Mohaghegh, Sajjad Baseri, Mohammad Hassan Kalantari, Rashin Giti, Seyed Ahmad Ghoraishian

**Affiliations:** 1 Dept. of Prosthodontics, School of Dentistry, Shiraz University of Medical Sciences, Shiraz, Iran; 2 Postgraduate Student, Dept. of Prosthodontics, School of Dentistry, Shiraz University of Medical Sciences, Shiraz, Iran

**Keywords:** Compressive strength, Marginal Adaptation, Zirconia, Sintering temperature

## Abstract

**Statement of the Problem:**

Increasing the sintering temperature is suggested by some manufacturers as a way to enhance the translucency of monolithic zirconia crowns. Meanwhile, its effect on the marginal fit and compressive strength of the restoration is not fully understood.

**Purpose:**

This study aimed to evaluate the effect of sintering temperature on the marginal fit and compressive strength of monolithic zirconia crowns.

**Materials and Method:**

In this *in vitro* study, thirty crowns of pre-sintered monolithic zirconia were milled and sintered in a special furnace at either 1450°C or 1550°C (n=15 per group). The marginal gaps were measured at 18 spots on the dies with a digital microscope. To evaluate the compressive strength, the specimens were cemented on brass dies by using conventional glass ionomer cement. Vertical load was applied by a universal testing machine until fracture. One-way ANOVA test was used to analyze the results (α=0.05).

**Results:**

The marginal gap was not significantly different between the two groups (*p*= 0.062). A significantly higher mean value of compressive strength was observed in crowns sintered at 1550°c (1988.27±635.09 N) than those sintered at 1450 °c (1514.27±455.11 N) (*p*= 0.026).

**Conclusion:**

Although increasing the sintering temperature would not affect the marginal gap of monolithic zirconia crowns, it could significantly improve the compressive strength of zirconia restorations.

## Introduction

Zirconia-based restorations are very popular due to their supreme strength, outstanding fracture resistance and excellent biocompatibility [ 1 - 3 ]. Zirconia ceramics exist in multiple crystalloid modes including monoclinic (room temperature up to 1170°C), tetragonal (1170-2370 °C) and cubic (2370°C to melting point). When zirconia cools down to room temperature, phase transformation from tetragonal to monoclinic happens to it, along with a 3%-5% volume expansion, which causes stresses through the material and can led to destruction [ 4 - 5 ]. 

Being highly opaque, zirconia cores are generally veneered with veneering porcelain. Clinically, the veneer is much more vulnerable to chipping and failure [ 6 ]. Aiming to decrease the costs and defeat the chipping problem, a nano-crystalline zirconia have been developed as yttria-stabilized tetragonal zirconia polycrystal (Y-TZP), with satisfactory optical and mechanical properties, which allows fabricating fixed dental prostheses of monolithic zirconia without veneering ceramic [ 2 , 7 ]. 

These restorative solutions have no porcelain overlay material to jeopardize the shear or to cause fracture. Moreover, they do not require specialized pressing techniques and equipment. Constructing mono-block restorations from zirconia could enhance the mechanical stability and extend the domain of applications. There are significant advantages for monolithic crowns including reduced fabrication time, cost-effectiveness, and elimination of the core-veneer interface [ 7 - 8 ], as well as more conservative preparation due to the absence of the veneer layer [ 9 ]. Nonetheless, the high opacity of such zirconia restorations interferes with their esthetics [ 10 ]. Besides changing the formulation, modifying the fabrication and sintering process of zirconia can enhance the translucency and consequently improve the appearance [ 11 ]. Each sintering parameter can strongly affect the properties of zirconia [ 12 ]. Studies about the relationship between the microstructure and mechanical properties of Y-TZP reported that the transformation toughening depends on the grain size of these ceramics [ 13 - 15 ]; which is, in turn, influenced by any alteration in sintering time and temperature [ 16 ]. 

Sintering temperature generally spans from 1400°c to 1600°c based on the manufacturer’s instructions. Some manufacturers suggest increasing the sintering temperature as a way to enhance the translucency of monolithic zirconia crowns. Higher sintering temperature and time results in larger grain size, which is more likely to experience stress-induced transformation to a balanced structure and consequently increase the material toughness. The maximum toughness is achieved close to 1µm grain size. Beyond this critical threshold, the material transforms from tetragonal to monoclinic phase spontaneously, and consequently, diminishes in stability [ 17 ]. Sintering temperature highly determines the grain densification and thus the mechanical properties of specimen [ 18 ]. It may also affect the marginal fit, since ceramics shrink toward their bulk to different extents when cooling from higher temperature to room temperature [ 19 ]. 

Several studies have inspected the effects of changing sintering parameters (temperature and time) on the grain size, translucency, and biaxial flexural strength of zirconia materials [ 11 - 12 , 18 - 19 ]; however, limited evidence exists about the effect of sintering temperature on the compressive strength and marginal discrepancy of monolithic zirconium [ 20 - 21 ]. Thus, the current investigation was conducted to figure out the influence of sintering temperature on the marginal adaptation and compressive strength of monolithic zirconia crowns. The null hypothesis was that sintering the monolithic zirconia crowns at higher temperature would not influence the marginal adaptation and compressive strength of the monolithic zirconia crowns.

## Materials and Method

**Figure 1:**
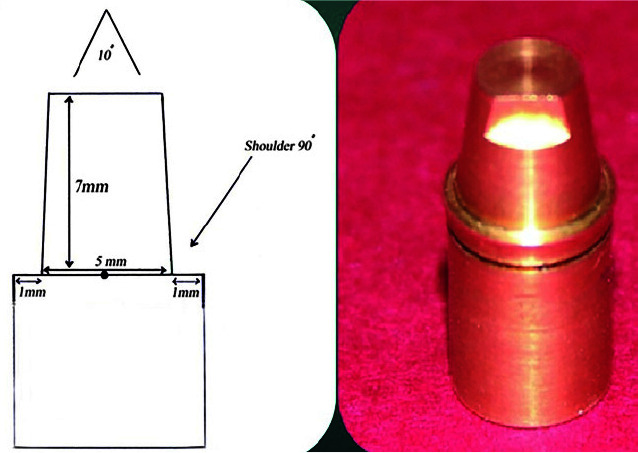
Master model

In this *in vitro* experimental study, 30 CNC (CNC350; Arix Co. Tainan, Hesin, Taiwan) machined standard brass master models (7×5 mm height × diameter) were constructed, with 90-degree 1-mm shoulder margin, 10-degree over all convergence angle (5-degree for each axial wall) and anti-rotational surface on the model (Figure 1).

To measure the marginal gap, 18 points were marked at 20-degree distances on a groove 3 mm beneath the finish line with a high-speed turbine (KaVo K9; KaVo Dental GmbH, Bismarckring, Biberach, Germany) and a small diamond fissure bur. The scan spray (Arti-Scan CAD-CAM Spray; Dr. Jean Bausch GmbH & Co KG, Oskar-Schindler, Köln, Germany) was applied to the dies and they were scanned using a 3 dimensional laser scanner (3Shape D810; 3Shape, Holmens Kanal, Copenhagen, Denmark). The scanned data were transported into the CAD software (3Shape CAD Design software; 3Shape, Holmens Kanal, Copenhagen, Denmark), in which a 30-µm cement space was assumed for the axial and occlusal surfaces of the abutment, with zero cement space at the finish line. 

The crowns thickness was considered 1 mm for the axial walls and 1.5 mm for the occlusal surface. All machined products were deliberated to be dimensionally 20% bigger than the real dies to gratify the sintering shrinkage. The design output was converted appropriately and conveyed to the processing machine to mill (inLab MC XL; Dentsply Sirona, Fabrikstrasse, Bensheim, Germany) 30 crowns from pre-sintered Y-TZP monolithic zirconia blanks (DD Bio ZX^2^, Dental Direkt, Industriezentrum, Spenge, Germany). They were categorized into two groups (n=15) to be sintered at either 1450°C or 1550°C (as recommended by the manufacturer) in a dental furnace (Programat S1; Ivoclar Vivadent, Schaan, Liechtenstein, Germany). The sintered crowns were examined and rejected in case of any imperfection.

**Figure 2:**
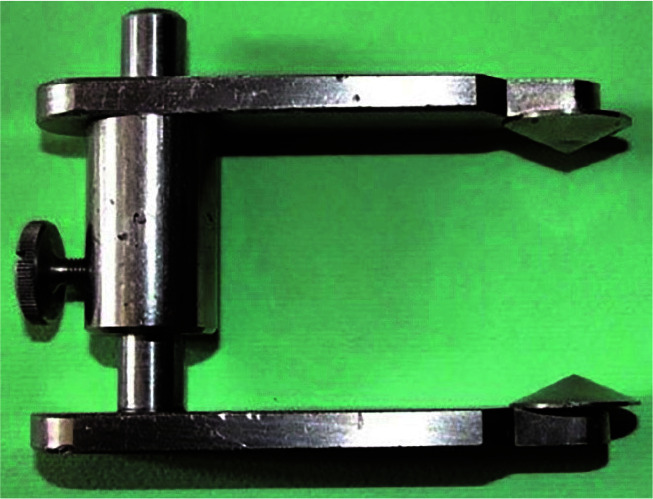
Holding instrument used for positioning of the specimens during the experiment

**Figure 3:**
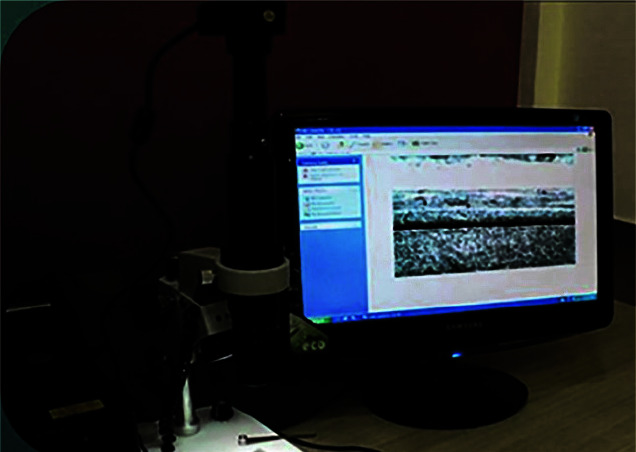
Image analyzing software

**Figure 4:**
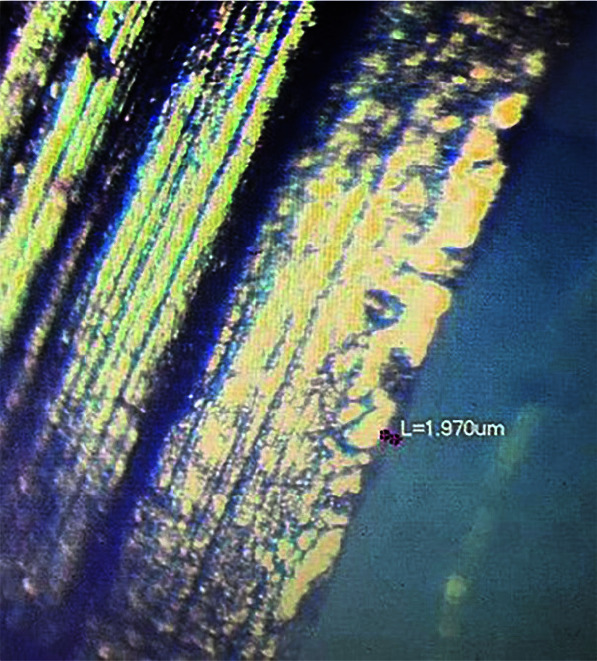
Marginal gap (230× magnification)

Having embedded the dies in resin blocks, a dental surveyor (Ney Dental Surveyor, Dentsply sirona, Degu-Dent GmbH, Rodenbacher Chaussee, Hanau-Wolfgang, Germany) was used to certify the long axis of the dies was perpendicular to the horizontal plane. The zirconia crowns were seated on the dies, using a specific holding device (Figure 2), with a uniform force of 15 N to warrant firm seating. By means of a light microscope (AM413FIT Dino-Lite Pro; Dino-Lite electronic corp., ChongHsin, Sanchong Dist., Taipei, Taiwan), images were taken and investigated at 230× magnification with an image analyzing software (DinoCapture 2.0, AnMo Electronics Corp., Tainan, Hsien, Taiwan). The marginal misfit was measured as the perpendicular line from the surface of the restoration margin to the outermost border of the preparation finish line (Figures 3 and 4).

**Figure 5:**
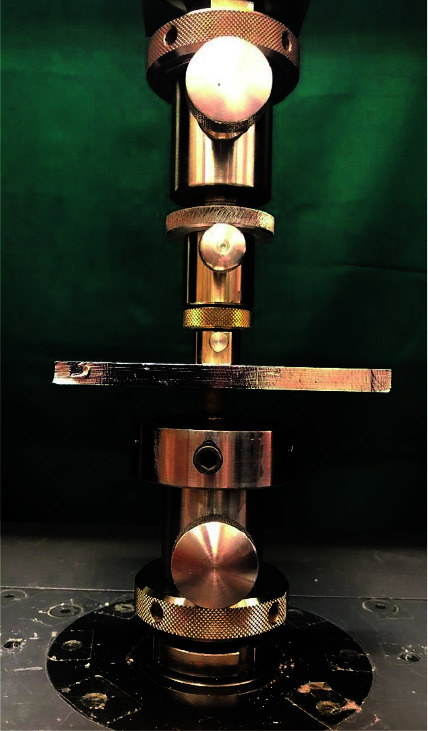
The crowns clamped in the holder of a universal testing machine and loaded vertically on the occlusal surface

The crowns were cemented with conventional glass ionomer cement (GC corp, Fuji I, Hongo, Bunkyoku, Tokyo, Japan) on the brass dies, which was already cleaned with steam and alcohol. Then, they were ﬁlled with the luting cement and loaded with firm finger pressure to allow the flow of excess cement. Thirty crowns were clamped in the holder of universal testing machine (ZwickRoell Z2.5; ZwickRoell, Kennesaw, GA, USA) and vertically loaded on the occlusal surface (Figure 5) with a thrust speed of 0.5 mm/min according to a previous study [ 22 ]. The minimum force leading to fracture was recorded by the computer software system that controlled the universal testing machine and completed the stress-strain diagram. 

**Figure 6:**
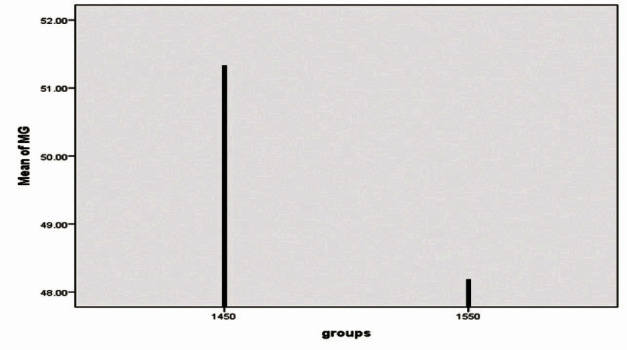
The mean marginal gap of the monolithic zirconia group sintered at 1450◦c was higher than those sintered at 1550◦c; however, the difference was not significant

Statistical analysis was performed using SPSS software (IBM SPSS Statistics, version 22.0; IBM Corp, NY, USA). The mean values and standard deviations were assessed, and one-way ANOVA test was applied to analyze the results (α=0.05).

## Results

The mean marginal misfit of the monolithic zirconia crowns sintered at 1450°C was 51.35±4.33μm and 48. 18±4.60μm for those sintered at 1550°C, which was not significantly different between the two groups (* p*= 0.062) (Figure 6). The mean compressive strength of the monolithic zirconia crowns sintered at 1550°C (1988.27 ±635.09 N) was significantly higher than that of those sintered at 1450°C (1514.27±455.11 N) (*p*= 0.026). The results of one-way ANOVA revealed that, while increasing the sintering temperature had no considerable impact on the marginal gap of monolithic zirconia crowns, it significantly improved the compressive strength (Table 1).

**Table 1:** Mean±SD of marginal gap (μm) and compressive strength (N) of the study groups

Sintering temperatures	1450°c	1550°c
Variables		
Marginal gap	51.35±4.33	48.18±4.60
Compressive strength	1514.27±455.11	1988.27±635.09

## Discussion

The absence of statistically significant difference between the marginal gaps of the test groups partially supports the null hypothesis, as increasing the sintering temperature did not affect the marginal discrepancy.

However, that part of null hypothesis addressing the compressive strength was rejected, since higher sintering temperature improved the compressive strength.

Different sintering temperatures are likely to alter the marginal fit as the ceramic materials shrink when cooling down to room temperature [ 19 ]. This shrinkage depends on various factors including the material composition, density, and factors of sintering procedure [ 18 - 22 ]. The resemblance of marginal fit values of different experimental groups in the present study may be due to the stability and strength of zirconia specimens. 

Various ranges of marginal gap are reported to be acceptable for all ceramic restorations. Previous studies reported a marginal gap range of 34 to 119μm or <120μm to be clinically acceptable [ 23 - 26 ]. Likewise, acceptable marginal discrepancies for CAD CAM crowns were reported to range from 50 to 100μm [ 25 ]. In the present study, the mean marginal misfit of monolithic zirconia was within clinically acceptable ranges for both groups of sintering temperatures. Moreover, the marginal gap was measured without cementing the crowns to preclude the variety of cementation procedure (applied force and the mixture viscosity) [ 26 ]. Numerous investigations demonstrated that cementation significantly increased the marginal misfit, depending on the luting agent [ 27 - 28 ].

The marginal discrepancy of restorations has been assessed with various methods, the most common of which are direct microscopic view and the replica method [ 29 - 30 ]. As a non-destructive straightforward technique, direct viewing evaluates the marginal fit by means of stereomicroscopes [ 31 ] and optical microscopes [ 26 ] along with an image analysis software, as used in the present study.

The current study also detected a direct relationship between the sintering temperature and compressive strength of monolithic zirconia crowns. Despite the narrow temperature range (100°C), the compressive strength significantly improved, substantiating the fact that even small sintering temperature variations can significantly affect the compressive strength of zirconia crowns.

In line with the present study, Ersoy *et al.* [ 18 ] noticed improved flexural strength of zirconia, fine structure, and densification following the concurrent increase of sintering temperature and decrease of sintering time. Examination of the crystal structure showed that all samples were totally sintered to the tetragonal stage and no conversion to monoclinic stage was distinguished. Similarly, Stawarczyk *et al.* [ 12 ] studied the influence of sintering temperature on the contrast ratio and biaxial flexural strength of zirconia discs. They achieved the highest flexural strength between 1400°C and 1550°C; however, Stawarczyk *et al.*oolllll [ 12 ] reported that the flexural strength declined over 1550°C due to immigration of yttrium to the grain boundaries. Tekeli and Erdogan [ 32 ] documented that higher sintering temperature and extended dwell time yielded greater grain size, and consequently, greater micropores, which diminished the mechanical properties of the material. It contradicted the present findings, which showed that higher sintering temperature enhanced the compressive strength. This difference can be attributed to the varying brands (and therefore composition) of zirconia used in both studies as well as the narrower temperature range examined in the present research.

Transformation toughening in zirconia ceramics depends on the grain size [ 13 - 15 ], which is, in turn, affected by the sintering temperature [ 16 ]. Although higher sintering temperature creates zirconia specimens with larger grains and consequently higher toughness, there is a threshold for the grain sizes beyond which tetragonal-monoclinic transformation and subsequently decrease of material stability is expected [ 17 ]. It seems that 1550°C is the critical sintering temperature; beyond which grain size larger than the critical size is obtained [ 5 , 33 - 34 ]. It was stated that larger grain size might enhance crack formation [ 35 ] and consequently decrease the mechanical strength of material. However, such a trend was not observed in the present study as the temperature was below the critical threshold. 

The failure load in complete crowns was reported to range from 980 to 1400 N [ 36 - 37 ]; while in the present study, the mean compressive strength of monolithic zirconia was 1514N (at 1450°c) and 1988N (at 1550°c). The present study assessed the compressive strength while the crowns were cemented on brass dies (not natural teeth) to control the similarity of factors like margin and crowns convergence. The elastic modulus and fracture strength of the crowns are not similar to natural teeth. Hence, the higher compressive strength may be attributed to the high elastic modulus of the supporting structure, and the subsequent overestimation of the clinical values. Some previous research showed that increasing the elastic modulus of surrounding assembly improved the crowns failure resistance [ 38 - 40 ]. 

The *in vitro* nature of this study did not allowed precise simulation of clinical conditions; thus, clinical studies are necessary to confirm *in vitro* findings. Studies are also required to investigate the influence of increasing the sintering temperature on other mechanical properties of zirconia ceramic restorations. This study examined only one zirconia brand, which restricts applying the findings for another zirconia brands with various grain sizes and compositions. Beside, only two sintering temperatures were investigated; while, there is an urge to assess the effect of higher sintering temperatures on the mechanical characteristic of zirconia restorations.

## Conclusion

Considering the restrictions of this *in vitro* study, it can be driven that increasing the sintering temperature would not considerably influence the marginal gap of monolithic zirconia crowns. However, it can significantly improve their compressive strength.

## Acknowledgment

This article was based on the thesis by Dr. Sajad Baseri in partial fulfillment of MSc. degree. The authors would like to thank the Vice-Chancellery of Research of Shiraz University of Medical Sciences for supporting this research study (Grant#18039). Appreciations are also expressed to Dr. Mehrdad Vosoughi from the Dental Research Development Center for his contributions in statistical analyses and Ms. Farzaneh Resole for proofreading, editing, and improving the English structure of this manuscript.

Vice-Chancellery of Research of Shiraz University of Medical Sciences (Grant#18039)

## Conflict of Interest

The authors declare no conflicting interest.
